# The Late Dr. T. D. Savill

**Published:** 1910-04-23

**Authors:** Harry Campbell, J. Henry Chaldecott, George Davis

**Affiliations:** Hon. Trustees to the Fund.; Hon. Trustees to the Fund.; Hon. Trustees to the Fund.


### Medical Letter-Box.

THE LATE DR. T. D. SAVILL.

Sirs,—In view of the valuable work which was done by the late Dr. Savill in connection with nervous diseases, a scheme has been inaugurated in furtherance of a wish, frequently expressed by him during his life, to found a scholarship at the West End Hospital for Diseases of the Nervous System.

A considerable sum has already been received, and any further donations from professional friends and others will be gratefully accepted. Contributions should be addressed to Savill Memorial Fund, c/o London County and Westminster Bank, 1 Connaught Street, Hyde Park, W.

We are, sirs, yours faithfully,
Harry Campbell,
J. Henry Chaldecott,
George Davis.
(Hon. Trustees to the Fund.)

